# Effect of Implantable Electrical Nerve Stimulation on Cortical Dynamics in Patients With Herpes Zoster–Related Pain: A Prospective Pilot Study

**DOI:** 10.3389/fbioe.2022.862353

**Published:** 2022-05-16

**Authors:** Haocheng Zhou, Rui Han, Li Chen, Zhen Zhang, Xiaobo Zhang, Jianlong Wang, Zuoliang Liu, Dong Huang

**Affiliations:** ^1^ Department of Pain, The Third Xiangya Hospital and Institute of Pain Medicine, Central South University, Changsha, China; ^2^ Hunan Key Laboratory of Brain Homeostasis, Central South University, Changsha, China; ^3^ Department of Orthopedics, The Third Xiangya Hospital, Central South University, Changsha, China; ^4^ Department of Critical Care Medicine, The Third Xiangya Hospital, Central South University, Changsha, China

**Keywords:** implantable nerve stimulation, spinal cord stimulation, peripheral nerve stimulation, neuromodulation, herpes zoster–related pain, cortical, EEG

## Abstract

Implantable electrical nerve stimulation (ENS) can be used to treat neuropathic pain caused by herpes zoster. However, little is known about the cortical mechanism underlying neuromodulation therapy. Here, we recorded a 16-channel resting-state electroencephalogram after the application of spinal cord stimulation (*n* = 5) or peripheral nerve stimulation (*n* = 3). The neuromodulatory effect was compared between specific conditions (active ENS versus rest). To capture the cortical responses of ENS, spectral power and coherence analysis were performed. ENS therapy achieved satisfactory relief from pain with a mean visual analog scale score reduction of 5.9 ± 1.1. The spectral analysis indicated that theta and alpha oscillations increased significantly during active neuromodulation compared with the resting state. Furthermore, ENS administration significantly increased frontal-frontal coherence in the alpha band. Our findings demonstrate that, despite methodological differences, both spinal cord and peripheral nerve stimulation can induce cortical alpha oscillation changes in patients with zoster-related pain. The dynamic change may, in part, mediate the analgesic effect of ENS on herpes zoster–related pain.

## 1 Introduction

Herpes zoster (HZ) is a common viral disease caused by the latent reactivation of the varicella-zoster virus and mainly affects the trigeminal nerve or dorsal root ganglia. The overall incidence of HZ has been estimated to be approximately 3.1 per 1,000 person-years in the last three decades in the United States (Insinga et al., 2005). Despite the common appearance of zoster rash, HZ patients may present with various phenotypes of abnormal sensations (e.g., itching, burning, hyperesthesia, and severe pain) in the infected dermatomes (Fashner and Bell, 2011). The application of antiviral therapy to relieve HZ-related pain at the initial diagnosis of HZ is crucial and may be combined with additional analgesic agents (Fashner and Bell, 2011). However, enduring HZ-related pain and greater pain intensity are associated with a higher risk of postherpetic neuralgia (PHN), which is characterized as a chronic status of neuropathic pain (Kost and Straus, 1996; Jung et al., 2004). PHN is well-known as the most common and severe complication following an HZ attack. Consequently, the development of PHN not only increases the burden on the public health system but also significantly reduces the quality of life of patients (Johnson et al., 2010). Thus, alternative treatment options are needed to control HZ-related pain.

A considerable amount of evidence has demonstrated that the implantable electrical nerve stimulation (ENS) device can provide sufficient relief from HZ-related pain (Harke et al., 2002; Vannemreddy and Slavin, 2011; Deer et al., 2014; Chen et al., 2017; Ni et al., 2021). For pain management, ENS can be classified into two approaches according to the site of the herpetic lesion: spinal cord and peripheral nerve stimulation. Limited data from case reports are available to support the use of deep brain stimulation for herpetic pain (Green et al., 2003; De Vloo et al., 2019). For acute or subacute HZ-related pain, both central and peripheral neuromodulation provides satisfactory pain control with the temporary implantation of a stimulator (Harke et al., 2002; Han et al., 2020; Wan and Song, 2020). For severe PHN cases, those who are intractable to conventional therapy, permanent implantation of an ENS device can be considered. However, even if the test electrode is positioned optimally, and the sensation of paresthesia completely covers the painful region, approximately 17–20% of patients may have a negative response (Kumar and Wilson, 2007; Wolter, 2014). Thus, a successful trial of ENS is required before the permanent implantation of a pulse generator. One major complication of ENS is the reduction in analgesic effect over time (Sparkes et al., 2010). Burst stimulation and high-frequency stimulation may attenuate the tolerance to ENS (Zeidman, 2018). However, the function of novel stimulation strategies requires further validation because of the lack of knowledge regarding the mechanism of action.

It is well established that the supraspinal mechanism plays an important role in the processing of pain signals (Bliss et al., 2016; Urien and Wang, 2019; Bannister and Dickenson, 2020). Recently, emerging evidence has demonstrated the association between cortical changes and the antinociceptive effect of spinal cord stimulation in rats with neuropathic pain (Koyama et al., 2018). Consistent with preclinical data, the spectrum power of the theta band is significantly altered in patients with failed back surgery syndrome (FBSS) who are treated with ENS therapy (Goudman et al., 2020). However, novel stimulation strategies at higher frequencies (ranging from 500 Hz to 30 kHz) alter the phenotype of cortical dynamics, which is not observed with conventional stimulation strategies (Goudman et al., 2020; Telkes et al., 2020). In addition to the spinal cord, a recent study has provided novel insights into the supraspinal mechanism induced by low-frequency stimulation of the peripheral nerves (Arendsen et al., 2021). To the best of our knowledge, few studies have examined the cortical effects of ENS in an HZ cohort. Therefore, this study aimed to evaluate cortical responses to ENS in patients with HZ-related pain.

## 2 Materials and Methods

### 2.1 Participants

This prospective observational study was approved by the Ethics Committee of The Third Xiangya Hospital, Central South University, China (S2020-552), and conducted in accordance with the Helsinki Declaration. Written informed consent was obtained from all subjects. The study was registered at chictr.org.cn (ChiCTR2100043097). Potential participants presented at the Department of Pain of The Third Xiangya Hospital with severe herpetic pain that was intractable to conventional therapy and who were scheduled to undergo ENS implantation between November 2020 and March 2021. The diagnosis of HZ was confirmed by the characteristic vesicular rash and dermatomal pain of the affected nerves. Age, sex, duration of infection, and location of HZ; preoperative, postoperative, and final follow-up visual analog (VAS) scores; date of ENS implantation and hospitalization; and ENS parameters were recorded systematically in one standard form.

### 2.2 Procedure of Peripheral Nerve Stimulation

**FIGURE 1:**
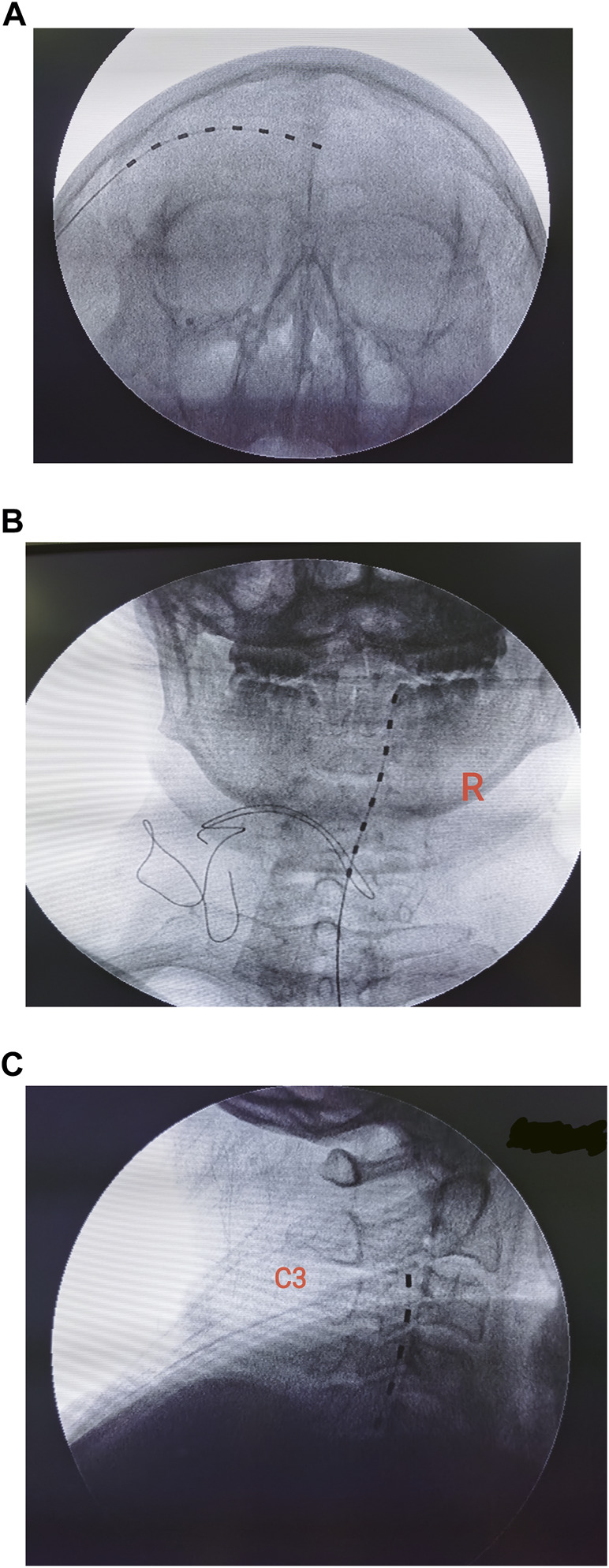
Placement of electrodes was confirmed by fluoroscopic imaging. **(A)** Implantation of the peripheral nerve stimulation device. **(B,C)** Position of cervical spinal cord electrode confirmed by anterior–posterior and lateral views.

Details of the surgical procedure have been described by Lerman et al. (2015) previously. Specifically, the head of the patient was turned contralateral to the surgical site in a supine position. Local anesthesia was administered with 1% lidocaine infiltrating 2 cm lateral to the orbit at the level of the supraorbital ridge and superficial temporal fascia. One Tuohy needle (14G) was applied to guide electrode implantation. The entry point was approximately 2 cm posterolateral to the junction of the frontal and zygomatic portions of the orbital rim. The guiding needle was advanced above the eyebrow along a semilunar path, cephalad to the orbicularis oculi, terminating slightly beyond the cranial midline. An eight-contact stimulation lead (model 3873; Medtronic, Minneapolis, MN, United States) was inserted through the Tuohy needle, overlying the supraorbital ridge as identified by fluoroscopic imaging (Figure 1A). The distal end of the lead was then connected to an extension multi-lead cable (Model: 355531; Medtronic, Minneapolis, MN, United States). To program the PNS, the external cable was plugged into a neurostimulator (Model 37022; Medtronic, Minneapolis, MN, United States).

### 2.3 Procedure of Spinal Cord Stimulation

The temporary implantation of the spinal cord stimulator was performed under fluoroscopic guidance with local anesthesia (Dong et al., 2017). The targeted spinal level of implantation was evaluated preoperatively according to the dermatomes of the HZ lesion. The patient was placed prone, and the paramedial approach was used to insert the Tuohy needle into the epidural space (Dong et al., 2017). The needle stylet was removed, and an eight-contact stimulation lead (model 3873; Medtronic, Minneapolis, MN, United States) was inserted through the Tuohy needle and advanced toward the targeted spinal segment under fluoroscopic imaging (Figures 1B,C). A test of the stimulation was required during implantation to ensure optimal coverage of the painful dermatomes. We only enrolled patients in the study who had only one electrode implanted. To avoid the potential migration of electrodes, patients were required to stay in bed for 48 h after surgery.

### 2.4 Experimental Protocol

**FIGURE 2:**
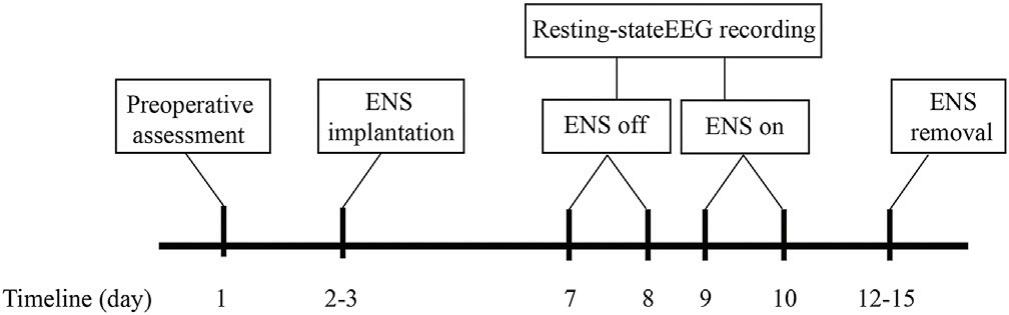
Schematic of the study protocol.

This study consisted of two hospital visits after the implantation of the ENS device. During each visit, a 5-min electroencephalogram was recorded at rest. The first EEG recording was acquired approximately 4–5 days after the ENS device implantation. The parameters for ENS were set in advance to ensure optimal coverage of the painful region and kept active during the first recording. The second visit took place on the next day after the initial EEG recording. To evaluate the cortical changes after ENS inactivation, one researcher (RH or ZZ) turned off the stimulator at least 30 min before the start of the subsequent EEG recording. A traditional low-frequency stimulation strategy (50 Hz) was selected for both SCS and PNS, with pulse widths ranging between 450 and 500 μs. The voltage of stimulation was adjusted to induce the sensation of paresthesia for pain relief in the affected regions. The duration of neurostimulator implantation was no more than 14 days to avoid potential infection based on the clinical routine at our center (Han et al., 2020). The flow chart of the study protocol is provided in Figure 2.

### 2.5 Electroencephalogram Recordings

The EEG recordings were conducted in a noise-free, temperature-controlled, and electrically shielded room. During the recording sessions, participants were instructed to stay silent and awake (with their eyes closed). For the acquisition of signals, we used an EEG electrode cap, which was connected to a 16-channel biosensing board (Cyton + Daisy, www.OpenBCI.com), with Cz and Fpz as the reference and ground electrodes, respectively. FP1, FP2, F3, F4, F7, and F8 electrode sites were classified as the frontal region (Table 1). C3 and C4 electrode sites were considered the central region; for occipital sites, electrodes were placed at O1 and O2; for parietal sites, electrodes were placed at P3, P4, P7, and P8; and for temporal sites, electrodes were placed at T7 and T8. The OpenBCI GUI software was used for data visualization and storage during the experiment. The sampling rate of the EEG signals was set at 128 Hz. The impedances of electrodes were evaluated and maintained as low as possible below 10 KΩ.

**TABLE 1:** Definition of the 16-channel electroencephalogram electrodes by regions of interest.

	Electrodes (channel number)
Frontal	FP_1_, FP_2_, F_3_, F_4_, F_7_, F_8_
Central	C_3_, C_4_
Parietal	P_3_, P_4_, P_7_, P_8_
Occipital	O_1_, O_2_
Temporal	T_7_, T_8_

### 2.6 Electroencephalogram Data Processing

Raw EEG data were extracted into MATLAB 2018b (MathWorks, Inc., Natick, MA, United States) for offline preprocessing using the open-source EEGLAB toolbox (Delorme and Makeig, 2004). All EEG data were visually examined to exclude artifacts and malfunctioning channels. Continuous EEG data were band-pass filtered (1–45 Hz), and the filtered data were then segmented into consecutive 2-s epochs. Amplitudes of epochs over ±80 μV were excluded from further analysis. Eye movement artifacts were identified and corrected by applying independent component analysis (Villafaina et al., 2019). Twenty artifact-free segments were included in the final data set for further analysis.

Analysis of the spectral power density of the brain was conducted using the fast Fourier transform algorithm with the “spectopo.m” function in EEGLAB. The data were banded into five physiological ranges: δ (delta, 0.5–4.0 Hz), θ (theta, 4.0–8.0 Hz), α (alpha, 8.0–13.0 Hz), β (beta, 13.0–30.0 Hz), and γ (gamma, 30.0–45.0 Hz). The spectra of individual channels were averaged across epochs for each patient. The spectral power density provided an output of signal strength in terms of μV^2^/Hz.

EEG coherence represents the degree of correlation between two electrodes at a specific frequency. The HERMES toolbox was used to compute the coherence between two frontal EEG electrodes FP2 and F7 (Niso et al., 2103). Mathematically, coherence was computed using the following equation:

$$COH_{xy}\left(f\right)={\left|K_{xy}\left(f\right)\right|}^{2}=\frac{{\left|S_{xy}\left(f\right)\right|}^{2}}{S_{xx}\left(f\right)S_{yy\left(f\right)}},$$

where *S*_*xy*_(*f*) denotes the cross-spectrum between signals *x*(*t*) and *y*(*t*) and their individual power spectral densities *S*_*xx*_(*f*) and *S*_*yy*_(*f*). The value of coherence ranged from 0 (no linear dependence) to 1 (maximum coherence). We calculated the estimated peak coherence and its frequency for the frontal oscillation of each patient (Akeju et al., 2014).

### 2.7 Statistical Analysis

Descriptive statistics was used to present the clinical information of participants. Variables are presented as means ± standard deviations. All EEG data were processed using MATLAB R2018b (MathWorks, Inc., Natick, MA, United States) and extracted into Prism v8 (GraphPad, San Diego, CA, United States) for further statistical analysis. A two-way repeated-measures analysis of variance (ANOVA) and *post hoc* multiple pairwise Bonferroni tests were used to compare spectral and coherence estimates at each sub-band under different conditions (ENS off versus ENS on). A *p* < 0.05 was considered statistically significant.

## 3 Results

### 3.1 Clinical Characteristics of Participants

**FIGURE 3:**
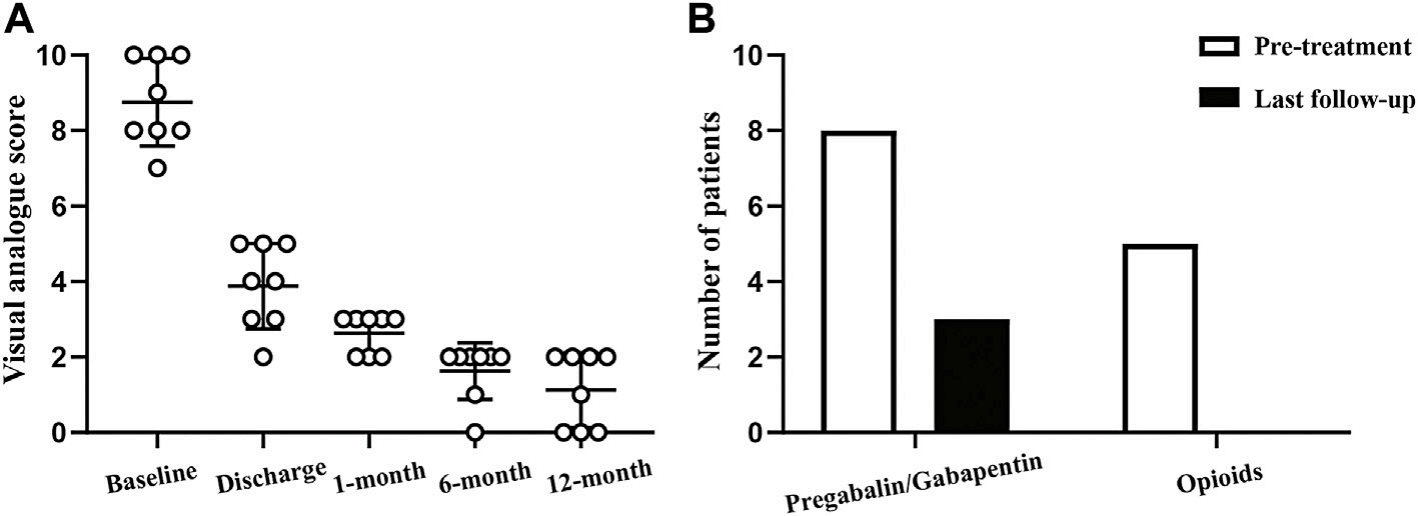
Follow-up of the therapeutic effect of ENS. **(A)** Changes in visual analog scale score before and after ENS therapy. **(B)** Analgesic medication use at baseline and final follow-up.

We initially recorded EEG signals from 11 patients pre- and post-surgery who had undergone ENS implantation. Three patients were excluded from further analysis because of poor-quality data. The average age of participants was 70.8 ± 4.1 years. All patients presented with subacute herpetic pain at admission, with the disease duration ranging from 1 to 3 months. Three participants experienced severe herpetic pain with ophthalmic involvement and agreed to undergo implantation of a peripheral stimulator, and five participants underwent spinal cord stimulation therapy. General pain relief was considerable with a mean VAS score reduction of 5.9 ± 1.1 at discharge. The overall pain severity decreased from 8.8 ± 1.1 to 3.9 ± 1.1 after neuromodulation treatment. No adverse effects (e.g., infection, hemorrhage, or tetrode migration) were reported in this study. The general clinical features of the enrolled patients are provided in Table 2 and Figure 3.

**TABLE 2:** General characteristics of patients.

Patient	Sex	Age	Duration	Location	VAS		Implantation	Hospitalization
		(years)	(months)		Baseline	Discharge	(days)	(days)
1	F	64	1	Op	10	3	13	19
2	F	70	1	Op	8	4	11	17
3	F	77	1	Op	10	5	10	14
4	F	75	1	C_2-5_	9	5	11	15
5	M	71	2	C_4-7_	10	5	10	17
6	F	71	1	T_2-4_	7	4	11	16
7	M	67	3	T_6-8_	8	3	10	13
8	F	71	1	L_2-4_	8	2	12	16

F, female; M, male; Op, ophthalmic; C, cervical; T, thoracic; L, lumbar; VAS, visual analog scale.

### 3.2 Spectral Analysis

#### 3.2.1 Global Changes in Spectral Power Density

**FIGURE 4:**
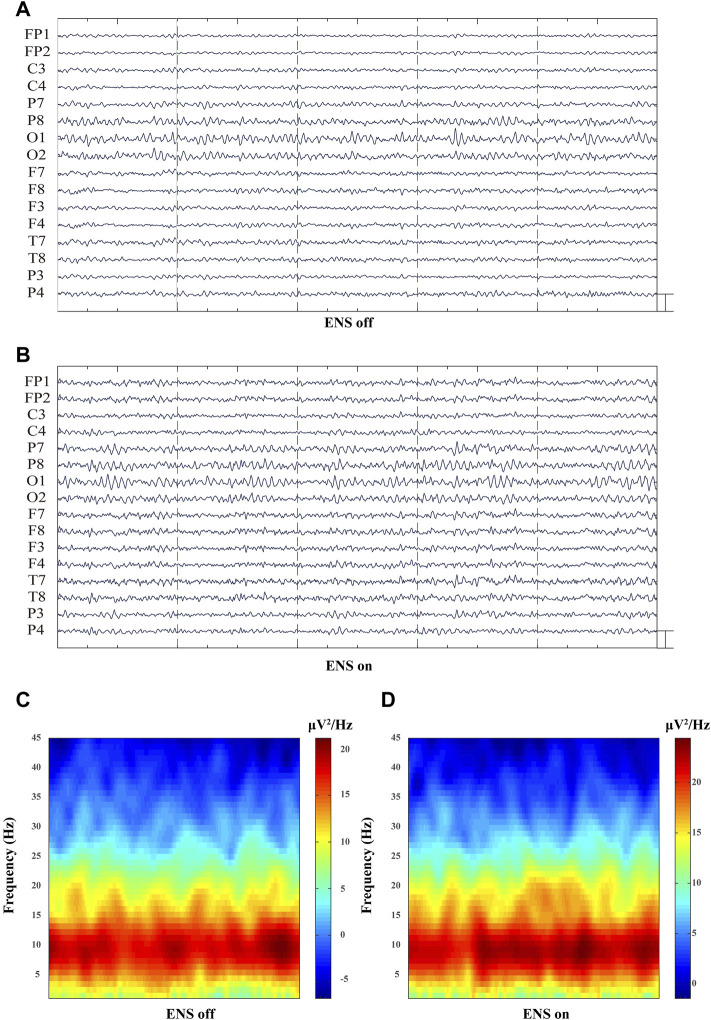
Representative multiple-channel EEG traces and time-domain spectrogram of baseline and ENS-induced EEG signatures. **(A)** Representative 10-s multiple-channel EEG traces at resting-state of ENS. **(B)** Representative EEG data during active ENS therapy. (Scale bar: 50 µV). **(C)** Baseline and **(D)** ENS-induced representative spectrogram.

**FIGURE 5:**
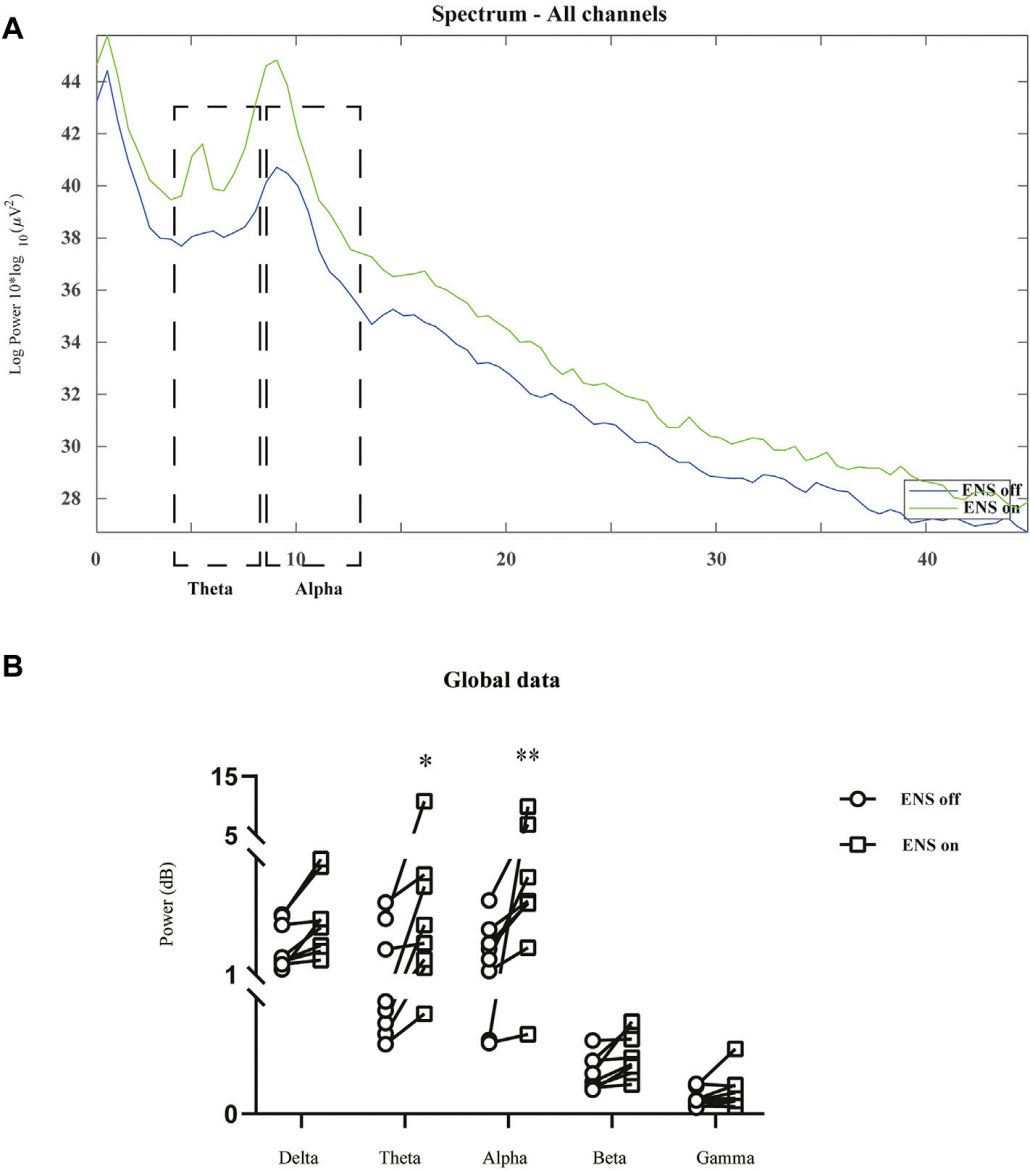
Comparison of neural oscillations between neuromodulation conditions using resting-state EEG recordings. **(A)** Grand average spectral power during resting-state (blue line) and active ENS (green line). **(B)** Significant enhancement of oscillatory activity was observed in the theta and alpha bands, tested using a repeated-measures two-way ANOVA with *post hoc* Bonferroni tests. *p < 0.05; **p < 0.01.

The spectral power density was estimated in 16 channels under the conditions of ENS on and ENS off (Figure 4). Initially, we computed the grand average spectral power by averaging across all channels and patients. The generalized enhancement of the spectrum was observed across all sub-bands with active neuromodulation (Figure 5A). In addition, ENS induced two significant peaks in the theta (4–8 Hz) and alpha (8–13 Hz) bands (Figure 5B).

#### 3.2.2 Electrical Nerve Stimulation–Induced Enhancement of Frontal Theta Oscillations

**FIGURE 6:**
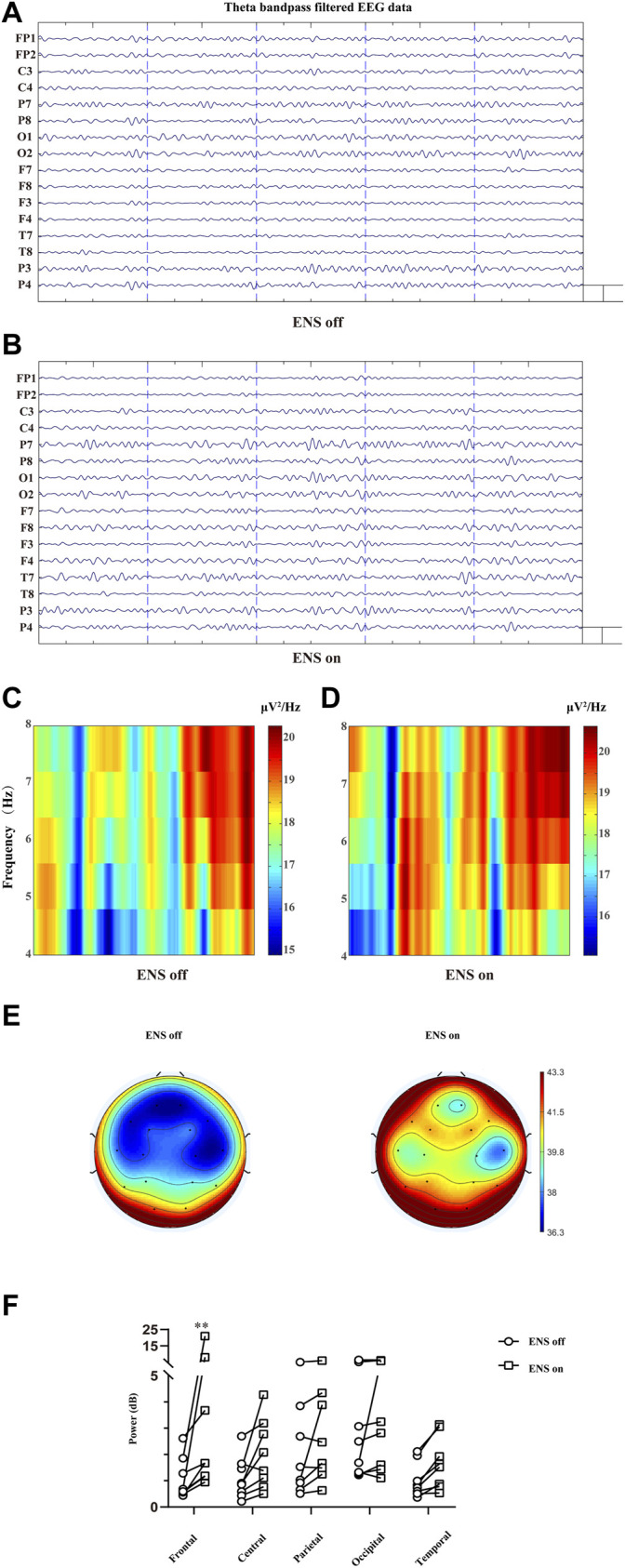
Cortical effect of ENS on theta activity. **(A,B)** Multiple-channel EEG data with theta band filtered at baseline and ENS activation. Representation of time–frequency domain spectrogram (4–8 Hz) at baseline **(C)** and ENS therapy **(D)**. **(E)** Topographic power spectral density maps in the theta (4–8 Hz) frequency band. **(F)** Theta oscillations increased significantly in the frontal region, tested using a repeated-measures two-way ANOVA with *post hoc* Bonferroni tests. *p < 0.05.

To investigate the spatial distribution of theta oscillations (Figures 6A,B), we computed the average spectral power of the theta band for each brain region of interest (Table 1). The overall theta band power showed an increasing trend after activation of the neurostimulator (Figures 6C-E). However, only the frontal region was associated with a significant enhancement of theta oscillations (*p* = 0.0016; Figure 6F).

#### 3.2.3 Enhanced Occipital Alpha Power With Electrical Nerve Stimulation

**FIGURE 7:**
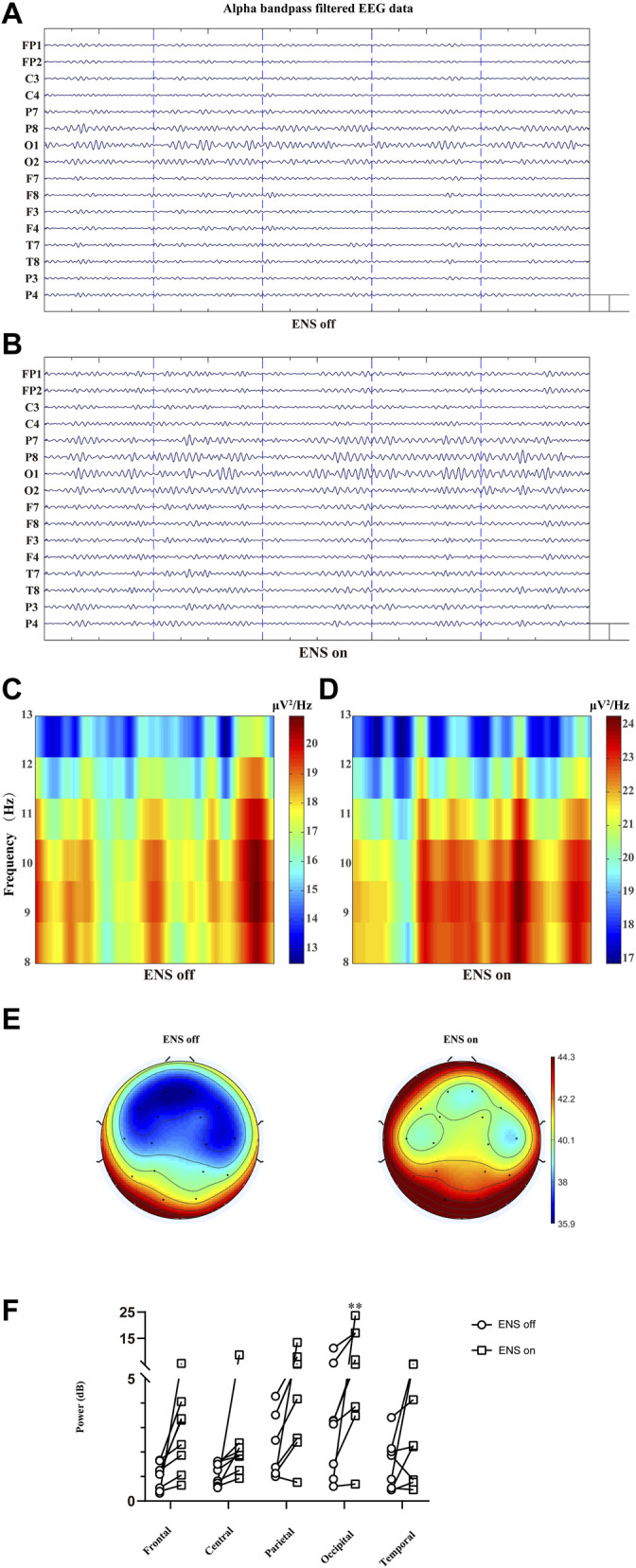
Comparison of the alpha oscillation (8–13 Hz) at different conditions of ENS, data traces **(A)** at baseline and **(B)** for neuromodulatory conditions. **(C,D)** Representative spectrogram of alpha activity at different states of ENS. **(E)** Topographic power spectral density maps in the alpha sub-band (8–13 Hz). **(F)** The power of alpha oscillations was significantly higher in the occipital region, tested using repeated-measures two-way ANOVA with *post hoc* Bonferroni tests. **p < 0.01.

In Figures 7A,B, we similarly compared the topographical distributions of alpha oscillation between two conditions (ENS off versus ENS on). The statistical analysis with Bonferroni correction (five brain regions and two ENS conditions) demonstrated significantly lower alpha spectral power density under resting state (Figure 7C) than active-state ENS (*p* = 0.0013; Figures 7D-F).

### 3.3 Coherence Estimation

**FIGURE 8:**
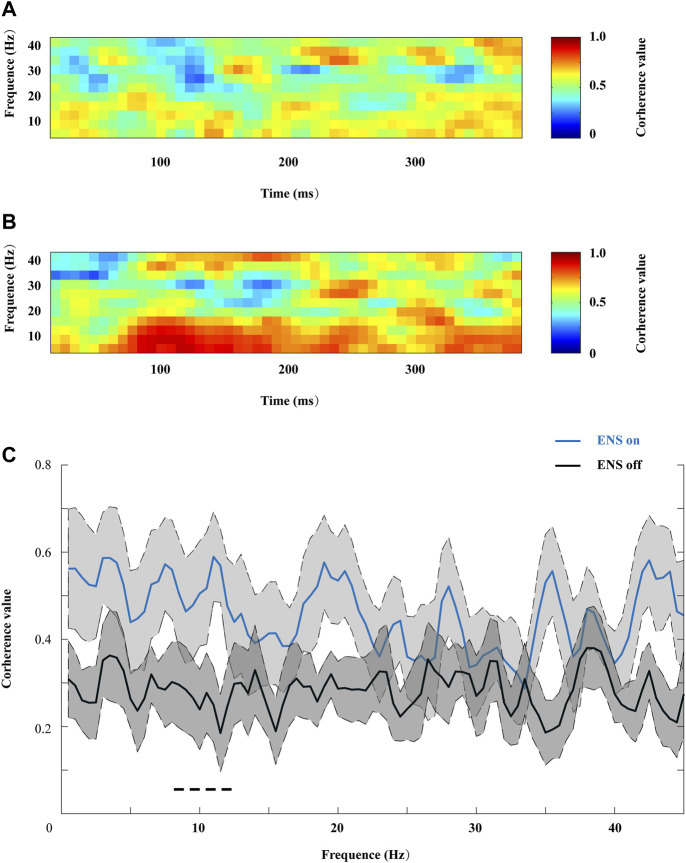
Representative coherograms within the 0–45 Hz frequency range, computed **(A)** from 400 ms before and **(B)** during ENS therapy. **(C)** Changes in coherence value were associated with ENS activation, and the peak coherence value of the alpha oscillation increased significantly under the ENS condition, tested using repeated-measures two-way ANOVA with *post hoc* Bonferroni tests. *p < 0.05.

We then compared coherence patterns during active- or resting-state ENS between the contralateral frontal sites (FP2 and F7), as shown in Figures 8A,B. ENS induced a generalized increase in coherence across all frequency ranges, and a significant decrease in coherence value was observed in the alpha sub-band when we turned off the stimulator (Figure 8C).

## 4 Discussion

In this study, we explored the cortical dynamics induced by ENS therapy in patients with herpetic-related pain. Our data suggested that the cortical features extracted from a herpetic population can distinguish activation of ENS from rest, regardless of the stimulation site. To treat trigeminal herpetic pain, the supraorbital and supratrochlear nerves are the most common electrode configurations (Winfree, 2020). This is consistent with clinical routine pain management for HZ ophthalmicus at our center (Han et al., 2020). In addition, spinal cord stimulation can be used to attenuate HZ pain in the truncal and upper and lower extremity regions (Yanamoto and Murakawa, 2012; Dong et al., 2017). One potential indication for temporary ENS implantation is the early onset of PHN, with a disease duration of fewer than 6 months (Yanamoto and Murakawa, 2012). In this study, the disease duration ranged from 1 to 3 months.

ENS is more widely applied to treat neuropathic pain in patients with FBSS than those with herpetic lesions. Although recent studies have explored the EEG patterns of ENS application in an FBSS population (Goudman et al., 2020; Telkes et al., 2020), to the best of our knowledge, this is the first study to examine cortical dynamics induced by ENS in patients with HZ-related pain. Previous data have suggested that higher stimulation dosage (high frequency) has a larger impact on the excitatory supraspinal pathway (Goudman et al., 2020; Telkes et al., 2020). Because of the short period of implantation, we did not compare EEG patterns under different stimulation parameters. In our study, a conventional stimulation frequency of 50 Hz, with a 500-μsec pulse width, was applied to produce comfortable paresthesia covering the painful region. Currently, evidence on the optimal stimulation strategy for HZ patients who undergo ENS therapy to control pain is limited. Thus, it is necessary to conduct a randomized controlled study in a larger sample to assess the effect of the stimulation strategy (conventional low frequency versus high frequency or burst stimulation) in an HZ cohort.

The supraspinal mechanism plays an important role in pain signal processing. Dysfunctional inhibitory or facilitatory descending pain modulation may contribute to the development of neuropathic pain syndromes that are caused by herpetic and diabetic diseases (Vanegas and Schaible, 2004; Baron, 2006). Recent neuroimaging studies have demonstrated structural and/or functional abnormalities in PHN populations in pain-related brain regions using functional magnetic resonance imaging (fMRI) and EEG approaches (Zhou et al., 2018; Li et al., 2020). In this study, we acquired EEG data to examine the real-time neuromodulatory effect of ENS. Although EEG provides millisecond temporal resolution of neural activity, determining the source of the signal is difficult because of insufficient spatial information. Thus, tools combining fMRI and EEG to examine the exact target of ENS in future studies would be helpful.

Previous data have proposed theta or alpha oscillatory activity as potential biomarkers of chronic pain with various etiologies, such as fibromyalgia, spinal cord injury, and breast cancer treatment (Tran et al., 2004; Van Den Broeke et al., 2013; Fallon et al., 2018). Specifically, patients with persistent pain following breast cancer show more alpha activity, and augmented theta oscillations are observed in fibromyalgia patients (Van Den Broeke et al., 2013; Fallon et al., 2018). Similarly, we found significant changes in theta and alpha activities during the activation of ENS when compared with the baseline, as shown in Figure 4. However, theta and alpha oscillations were shown to increase when high-dose, but not conventional, ENS was applied to an FBSS cohort (Goudman et al., 2020; Telkes et al., 2020). The inconsistencies in cortical responses may arise because of different etiologies of neuropathic pain. Therefore, it is necessary to compare dynamic changes in ENS due to different stimulation parameters in an HZ population.

After identifying increased theta and alpha oscillations, we further investigated changes in frontal connectivity induced by ENS therapy. Patients exhibited greater contralateral frontal connectivity when ENS was activated, as demonstrated by the peak coherence value in the alpha band (Figure 8). However, ENS has been shown to significantly decrease frontal-frontal connectivity in patients with disorders of consciousness (Bai et al., 2017). In the chronic pain state, enhanced frontal connectivity at theta (4–8 Hz) and gamma (>60 Hz) frequencies has been reported using state-of-the-art EEG analysis (Ta Dinh et al., 2019). Thus, it is unsurprising that both pain stimuli and pain relief increase the connection between different brain regions. Similarly, attention to pain stimuli increases the functional connectivity between critical pain-related brain regions (Liu et al., 2011a; Liu et al., 2011b). Therefore, we speculate that ENS therapy shares similar supraspinal mechanisms that underlie subject-driven (i.e., top-down) modes of attentional pain modulation (Hauck et al., 2015) to provide pain relief for HZ patients.

The primary limitation of this study is that we did not examine the EEG patterns of different ENS approaches, which was mainly because of the relatively small sample size. We plan to compare cortical responses between different ENS approaches in a future study with a larger cohort. In addition to the implantation site, stimulation frequency and other parameters may also affect therapeutic and cortical effects. Thus, it is necessary to conduct a randomized controlled study to determine the optimal stimulation strategy and the mechanisms involved.

## 5 Conclusion

Our data suggest that ENS therapy can affect alpha oscillations in patients with HZ-related pain despite differences in methodology. The dynamic change, in part, may be associated with the analgesic effect of ENS in patients with HZ-related pain.

## Data Availability

The raw data supporting the conclusion of this article will be made available by the authors, without undue reservation.
